# Case Report: Similar STR profiles in non-twin siblings complicating chimerism analysis after allogeneic hematopoietic stem cell transplantation

**DOI:** 10.3389/fmed.2026.1863294

**Published:** 2026-06-16

**Authors:** Amro A. Filfilan, Mohieldin Elsayid, Meaad Almowaled, Naif S. Sannan, Waseem Alzamzami, Doaa Alamoudi, Modhi Khamis, Dania A. Monagel

**Affiliations:** 1 Clinical Laboratory Sciences Program, College of Applied Medical Sciences, King Saud Bin Abdulaziz University for Health Sciences, Jeddah, Saudi Arabia; 2 King Abdullah International Medical Research Center, Jeddah, Saudi Arabia; 3 Ministry of National Guard-Health Affairs, King Abdulaziz Medical City, Jeddah, Saudi Arabia; 4 Department of Medical Laboratory Technology, Faculty of Applied Medical Sciences, University of Tabuk, Tabuk, Saudi Arabia; 5 College of Medicine, King Saud Bin Abdulaziz University for Health Sciences, Jeddah, Saudi Arabia; 6 Department of Pediatric Oncology, Ministry of National Guard-Health Affairs, King Abdulaziz Medical City, Jeddah, Saudi Arabia

**Keywords:** allogeneic hematopoietic stem cell transplantation, case report, chimerism monitoring, consanguinity, digital PCR, fanconi anemia, next-generation sequencing, short tandem repeat profiling

## Abstract

Fanconi anemia (FA) is an inherited bone marrow (BM) failure syndrome for which allogeneic hematopoietic stem cell transplantation (allo-HSCT) remains the only curative option. Short tandem repeat (STR) profiling is the reference molecular method for post-transplant chimerism monitoring, but its analytical performance depends on the presence of informative allelic differences between donor and recipient. We report a 3-year-old boy with FA carrying a homozygous FANCA c.2778 + 1G > A variant who underwent allo-HSCT from a human leukocyte antigen (HLA)–matched non-twin sibling donor. Pre-transplant STR analysis using two independent multiplex systems (PowerPlex^®^ 16 and PowerPlex^®^ ESI 16 Fast) revealed near-complete donor–recipient genetic concordance, with only a single informative autosomal locus (D13S317) available for chimerism tracking. Despite this analytical limitation, the patient achieved neutrophil engraftment on day +13 and platelet engraftment with sustained trilineage recovery through day +1376 (approximately 3.75 years post-transplant). Serial post-transplant STR monitoring demonstrated absence of the recipient-specific allele at D13S317, consistent with full donor-derived hematopoiesis and corroborated by independent assay replication and the clinical engraftment course. To our knowledge, this is among the first reported cases of single-locus STR informativity complicating post-HSCT chimerism analysis in a pediatric FA patient from a consanguineous population. In populations with high consanguinity, this case demonstrates a rare but actionable limitation of standard STR-based chimerism monitoring in non-twin siblings. It underscores the value of pre-transplant verification of donor–recipient STR informativity, predefined escalation to high-resolution alternatives such as digital PCR or next-generation sequencing–based chimerism analysis when fewer than two informative loci are identified, and cautious reporting language when molecular informativity is constrained.

## Introduction

Fanconi anemia (FA) is a rare inherited BM failure syndrome characterized by congenital anomalies, progressive pancytopenia, chromosomal instability, and a markedly elevated lifetime risk of myelodysplasia, acute myeloid leukemia, and solid tumors (1, 2). Allogeneic hematopoietic stem cell transplantation (allo-HSCT) remains the only curative option for the hematological manifestations of the disease, with human leukocyte antigen (HLA)–matched sibling donors associated with the most favorable transplant outcomes (2–4). Post-transplant chimerism monitoring is essential for documenting donor engraftment and for the early detection of graft failure, mixed chimerism, or relapse.

Short tandem repeat (STR) profiling is the reference molecular method for chimerism monitoring after allo-HSCT. Owing to their high heterozygosity, robust amplification on capillary electrophoresis platforms, and inter-laboratory reproducibility, multiplex STR assays remain the standard for quantifying donor–recipient chimerism and for detecting engraftment, mixed chimerism, and impending relapse (5, 6). Their analytical performance, however, depends on the presence of informative allelic differences between donor and recipient. Because full siblings share approximately half of their alleles by descent, the probability that two non-twin siblings will exhibit a complete match across all 15 standard autosomal STR loci has been estimated at approximately 1 in 5.8 million pairs, while a near-complete 14-of-15 match occurs in approximately 1 in 137,000 pairs (7, 8). In populations with high rates of consanguineous marriage, this allele-sharing baseline is shifted further upward by increased identity-by-descent. Saudi Arabia has reported consanguinity rates of 42–57%, and broader Middle Eastern genomic data document elevated homozygosity and extended runs of identity-by-descent in endogamous populations (9, 10).

What is unique about the present case is the convergence of three features rarely described together in the published literature: a pediatric FA recipient, an HLA-matched non-twin sibling donor, and pre-transplant STR concordance reduced to a single informative autosomal locus. Reports addressing similarly extreme STR concordance have so far come predominantly from the forensic-genetic literature (8, 11), and explicit guidance for transplant laboratories encountering such pairs is lacking. We use this case to characterize the analytical implications for STR-based chimerism monitoring under conditions of single-locus informativity, to compare and contrast with the small published literature, and to outline a practice-oriented escalation pathway.

## Materials and methods

### Ethical approval

The study was conducted in accordance with the Declaration of Helsinki and approved by the King Abdullah International Medical Research Center (IRB No. 00000127025; Study No. NRJ25/031/1). Written informed consent for publication was obtained from the patient’s legal guardians.

### Sample collection

BM aspirates were obtained from the recipient before transplantation, from the HLA-matched sibling donor before stem cell harvest, and from the recipient on post-transplant days +352 and +372. Genomic DNA was extracted using the QIAamp DNA Mini Kit (QIAGEN, Hilden, Germany) according to the manufacturer’s instructions. Peripheral blood samples and complete blood count (CBC) results were obtained as part of routine clinical evaluation at the National Guard Health Affairs (NGHA) Clinical Laboratory, Jeddah, Saudi Arabia.

### Recovery definitions

CBC results were used to assess engraftment kinetics and long-term hematological recovery. Neutrophil engraftment was defined as the first of three consecutive days with absolute neutrophil count (ANC) ≥ 0.5 × 10^9^/L. Platelet engraftment was defined as the first of seven consecutive days with platelet count ≥ 20 × 10^9^/L, sustained without platelet transfusion support.

### STR analysis and chimerism assessment

STR profiling was performed at an accredited reference laboratory (Bioscientia, Ingelheim, Germany) using two independent multiplex PCR systems run in parallel. The PowerPlex^®^ 16 System (Promega, Madison, WI, USA) co-amplifies 15 autosomal loci (D3S1358, TH01, D21S11, D18S51, D7S820, D16S539, vWA, D8S1179, FGA, D5S818, CSF1PO, D13S317, TPOX, Penta D, and Penta E) together with the sex-determining Amelogenin locus. The PowerPlex^®^ ESI 16 Fast System (Promega) was used in parallel as an independent multiplex chemistry to provide European Standard Set–compliant confirmatory profiling and to cross-verify allele calls at the overlapping autosomal STR loci. STR genotyping for both systems was performed according to the manufacturer’s validated protocol using approximately 0.5 ng of genomic DNA per reaction, with inclusion of positive and no-template negative controls in each run. Both multiplex assays were performed on the same DNA extract from each timepoint to enable cross-platform confirmation of allele calls. Amplicons were resolved by capillary electrophoresis on the Applied Biosystems™ 3500xL Genetic Analyzer, and allele calls were generated in GeneMapper^®^ ID v3.2 (Applied Biosystems) against the Promega allelic ladder.

Allele calls were retained only if they met the laboratory’s validated quality criteria: a minimum majority-allele signal of 1,800 RFU and a minimum minority-allele signal of > 50 RFU per the laboratory’s standard operating procedure; heterozygote peak height ratio ≥ 60%; n-4 stutter ratios within the locus-specific filter (typically ≤ 10–15% for tetranucleotide loci); absence of pull-up, dye blob, and spectral artifacts; and reproducibility across both multiplex systems. Reagent blanks and Promega 2800M control DNA were processed with every batch. These thresholds yield a calculated minimum detection limit of 2.7% [50 ÷ (1,800 + 50) × 100], within the 1–5% range reported for capillary STR chimerism assays; achievable signals up to 30,000 RFU allow substantially better realized sensitivity. This sensitivity applies only at loci where donor and recipient alleles differ; at concordant loci, mixed chimerism is undetectable in principle. In the present case, the 2.7% detection limit was therefore relevant only at D13S317, the single informative locus. Under single-locus informativity, qualitative donor-versus-recipient discrimination is preserved, but the practical limit of detection for low-level (≤5%) mixed chimerism is impaired by loss of cross-locus redundancy and widening of the single-locus confidence interval. Orthogonal high-resolution chimerism platforms (digital PCR, NGS-based SNP chimerism) were not performed during the monitoring period.

Pre-transplant donor and recipient STR profiles were compared to identify informative loci. Post-transplant specimens were evaluated for disappearance of recipient-specific alleles and concordance with donor allelic patterns. Because donor and recipient differed at only one informative autosomal locus, low-level mixed chimerism below the analytical sensitivity of conventional STR analysis could not be reliably excluded. Therefore, molecular findings were interpreted in conjunction with sustained hematologic recovery and long-term clinical follow-up.

## Results

### Patient presentation and baseline characteristics

A 3-year-old boy born to first-cousin consanguineous Saudi parents was referred for evaluation of thrombocytopenia (platelet count 30 × 10^9^/L) with easy bruising and gingival bleeding. The neonatal history was notable for low birth weight and poor feeding. On examination, the patient had failure to thrive, short thumbs, café-au-lait macules, and a soft systolic murmur. Renal scintigraphy demonstrated an ectopic solitary left kidney with preserved global function: normalized glomerular filtration rate (GFR) was 95.33 mL/min on the anterior view and 42.39 mL/min on the posterior view, with a geometric mean of 63 mL/min.

Initial laboratory evaluation showed hemoglobin (Hb) 11.5 g/dL, white blood cell count (WBC) 10.2 × 10^9^/L, neutrophil count 4.8 × 10^9^/L, platelet count 34 × 10^9^/L, and mean corpuscular volume 103 fL. Hb electrophoresis demonstrated HbA 63.0% and HbF 35.6%. BM examination showed hypocellularity without an excess of blasts, with reduced megakaryocytes accounting for the thrombocytopenia. Flow cytometry and conventional cytogenetic analysis revealed no clonal evolution. Chromosomal fragility testing was positive, and whole-exome sequencing identified a homozygous pathogenic splice-site variant (c.2778 + 1G > A) in intron 28 of the FANCA gene, confirming FA.

During the month preceding transplantation, progressive marrow failure was documented (Table 1): mean hemoglobin 10.14 g/dL, hematocrit 28.95%, white blood cell count 0.96 × 10^9^/L, ANC 0.22 × 10^9^/L, and platelet count 45.17 × 10^9^/L. An HLA-matched sibling donor was identified after extended family typing. The patient subsequently underwent allogeneic BM transplantation from his HLA-matched sibling donor following a fludarabine-based reduced-intensity conditioning regimen, with standard graft-versus-host disease prophylaxis according to institutional protocols for pediatric FA. The conditioning was tolerated without unexpected toxicity, and no adverse events required modification of the planned regimen.

**TABLE 1:** Baseline hematological parameters obtained 1 month prior to allogeneic hematopoietic stem cell transplantation.

Parameter	Mean (range)	Reference range
Hematocrit (%)	28.95 (20.3–35.0)	34.0–45.0
Hemoglobin (g/dL)	10.14 (7.3–12.1)	11.0–14.5
RBC count ( × 10^12^/L)	3.59 (2.4–4.4)	3.80–5.60
MCV (fL)	81.26 (76.3–87.7)	77–90
WBC count ( × 10^9^/L)	0.96 (0.1–3.4)	4.0–12.0
ANC ( × 10^9^/L)	0.22 (0.02–0.54)	2.00–7.50
Platelet count ( × 10^9^/L)	45.17 (9–92)	150–450

ANC, absolute neutrophil count; MCV, mean corpuscular volume; RBC, red blood cell; WBC, white blood cell.

### STR profile reveals extensive donor-recipient allelic concordance

The pre-transplant donor and recipient STR profiles were complete, of high quality, and concordant across both multiplex platforms. Complete genotypes were obtained for all loci. Comparison of the two profiles disclosed identical genotypes at 14 of the 15 autosomal STR loci, with discrimination preserved only at D13S317 (recipient 8,11; donor 11,12) (Table 2). Sample identity was independently verified using a separately obtained donor specimen, excluding sample exchange, contamination, and platform artifact, and supporting the interpretation that the near-identical profile reflected true identity-by-descent between non-twin siblings of consanguineous parents.

**TABLE 2:** Summary of STR genotypes across all samples.

Locus	Pre-Tx Recipient (J9905/23)	Donor (J9907/23)	Post-Tx D+352 (J9906/23)	Post-Tx D+372 (J9916/23)
Amelogenin	X, Y	X,Y	X,Y	X,Y
D3S1358	17, 18	17,18	17,18	17,18
TH01	7, 9	7,9	7,9	7,9
D21S11	30, 32.2	30,32.2	30,32.2	30,32.2
D18S51	12, 15	12,15	12,15	12,15
Penta E	7, 15	7,15	7,15	7,15
D5S818	10, 12	10,12	10,12	10,12
D13S317	**8, 11**	**11,12**	**11,12**	**11,12**
D7S820	8, 10	8,10	8,10	8,10
D16S539	11, 13	11,13	11,13	11,13
CSF1PO	10, 11	10,11	10,11	10,11
Penta D	10, 12	10,12	10,12	10,12
vWA	15, 17	15,17	15,17	15,17
D8S1179	12, 19	12, 19	12,19	12,19
TPOX	8, 12	8, 12	8,12	8,12
FGA	19, 23	19, 23	19,23	19,23

Bold row highlights the only informative locus, D13S317, where the pre-transplant recipient (8, 11) and donor (11, 12) differ. Post-transplant samples on day +352 and day +372 carry the donor-derived 11,12 genotype, with loss of the recipient-specific allele 8. Differential alleles between the pre-, post-transplant patient, and donor.

### Post-transplant STR Findings, chimerism assessment, and hematological recovery

Post-transplant chimerism monitoring was scheduled at days +352 and +372, with continuous clinical and hematological surveillance throughout the post-transplant course. Both post-transplant STR samples showed concordance with the donor allelic patterns, including disappearance of the recipient-specific allele 8 at D13S317 (Table 2). Electropherograms were superimposable in peak height and allele position between the donor and both post-transplant samples, with no emergent novel peaks and complete absence of the recipient-specific allele at D13S317. Within the analytical limitations described above, these findings are consistent with donor-derived hematopoiesis at the time points assayed. Because only one informative locus was available, the molecular result is reported as consistent with donor-derived hematopoiesis rather than as a quantitatively certified exclusion of ≤5% recipient signal.

**FIGURE 1:**
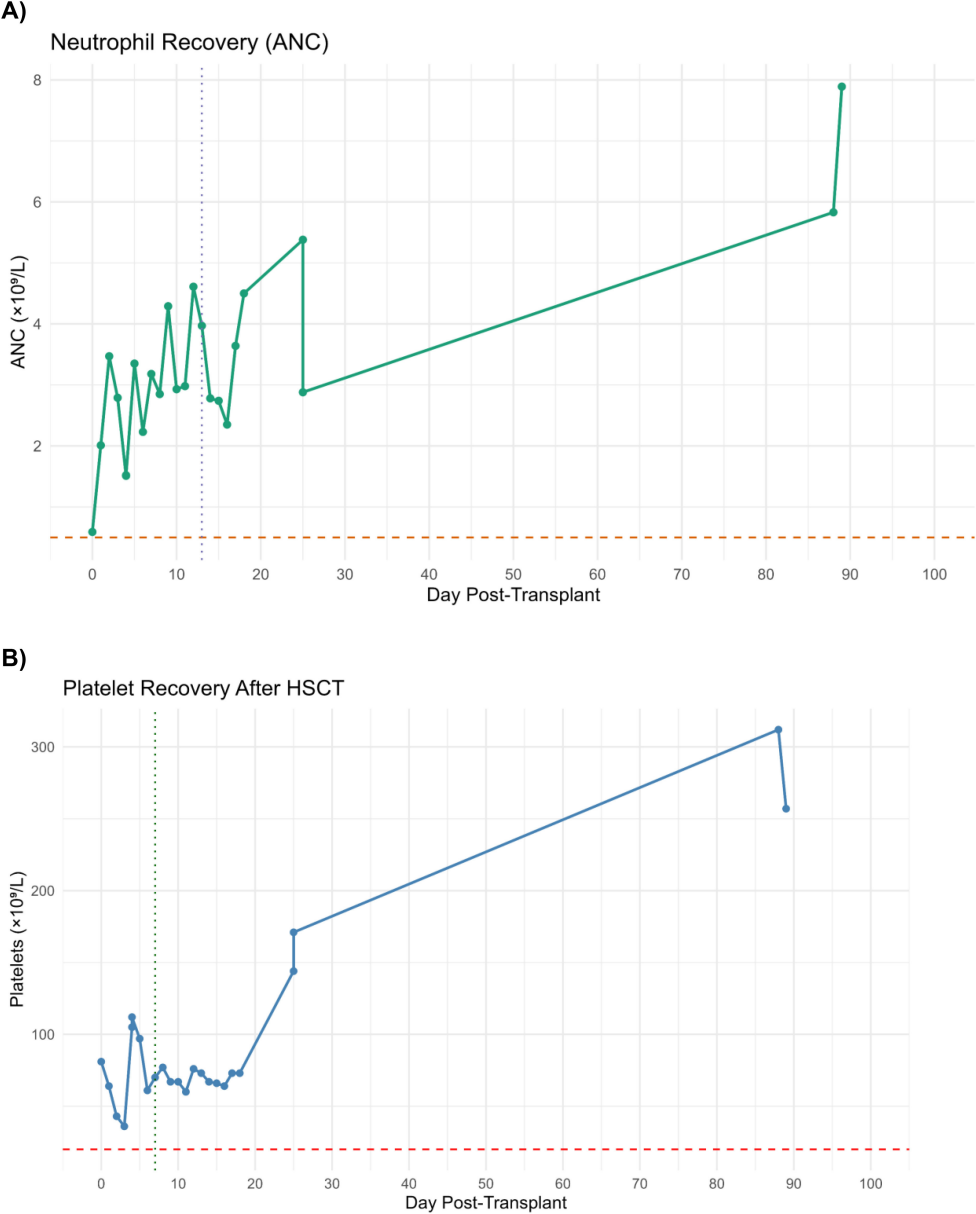
Hematological recovery following allogeneic HSCT. **(A)** Absolute neutrophil count (ANC, × 10^9^/L) monitored over the first 100 days post-transplant. The dashed horizontal line at 0.5 × 10^9^/L indicates the neutrophil engraftment threshold; engraftment was achieved on day +13. **(B)** Platelet count ( × 10^9^/L) recovery following allogeneic HSCT. The dashed horizontal line at 20 × 10^9^/L indicates the platelet engraftment threshold; transfusion independence was achieved with platelet counts ≥ 20 × 10^9^/L for seven consecutive days.

Engraftment was evaluated using the standardized criteria defined in Methods. Neutrophil engraftment was achieved on day +13, with sustained recovery thereafter (Figure 1A). Platelet engraftment was achieved with transfusion independence and platelet counts ≥ 20 × 10^9^/L for seven consecutive days (Figure 1B). Long-term follow-up demonstrated stable trilineage hematopoiesis through day +1376 (approximately 3.75 years post-transplant), with no evidence of graft failure, relapse, or secondary clonal evolution (Supplementary Table 1). The convergence of donor-pattern STR concordance, sustained trilineage recovery, and absence of clinical or laboratory features of graft dysfunction over more than three and a half years of follow-up provides multimodal evidence of durable donor-derived hematopoiesis. There were no adverse or unanticipated events related to the chimerism monitoring strategy. Throughout the post-transplant course, the family reported satisfaction with the frequency and clarity of communication regarding monitoring results. With stable trilineage recovery, the patient has returned to age-appropriate developmental and educational activities and the family has consented to publication of this case in the hope that it will inform the care of other children in similar consanguineous family settings.

## Discussion

We describe a case in which near-complete pre-transplant STR concordance between a Fanconi anemia recipient and his HLA-matched non-twin sibling donor reduced the informativity of standard chimerism monitoring to a single autosomal locus, a scenario that is rare, analytically instructive, and clinically actionable. The established population-genetic rarity of complete or near-complete STR matches between non-twin siblings has been documented in the forensic literature (7, 8), yet published reports of such concordance in the transplant-monitoring context remain scarce. Bandah-Rozenfeld et al. recently described two brothers whose autosomal STR profiles were nearly identical, requiring additional Y-STR and SNP analyses for forensic discrimination (11); earlier forensic series similarly emphasized the limits of standard 15-locus panels in highly related individuals (8). In contrast, the clinical implications of such concordance for post-HSCT chimerism monitoring have been only sparsely addressed; routine chimerism cohorts from low-consanguinity populations (3, 5, 6) have generally not encountered this scenario. To our knowledge, this is among the first reports documenting its consequences in a pediatric Fanconi anemia patient, extending the forensic literature into the transplant-monitoring setting.

When only a single informative locus is available, qualitative discrimination of full donor versus full recipient profiles is preserved, but the ability to detect low-level ( ≤ 5%) mixed chimerism is substantially impaired because cross-locus redundancy, used to distinguish true minor signal from stutter and noise, is lost. Several technologies overcome this ceiling: expanded STR panels (21–27 loci) offer an immediate capillary-compatible escalation, though they remain susceptible to the same identity-by-descent; indel panels exploit short biallelic polymorphisms with minimal stutter; SNP-based chimerism assays interrogate hundreds of biallelic markers with sensitivities of approximately 0.1–0.5% and demonstrated utility in risk stratification after allo-HSCT (12); and dPCR provides absolute quantification at 0.01–0.1%, independent of multi-locus STR informativity. These are clinically validated options whose adoption is particularly compelling in transplant programmes serving consanguineous populations.

This report leverages two independent multiplex STR systems on independently collected specimens, excluding platform artifact and sample swap, combined with extended longitudinal follow-up through day +1376 and serial CBC surveillance (Supplementary Table 1). The durable trilineage recovery over nearly 4 years, with no graft failure, relapse, or secondary clonal events, demonstrates the clinical compatibility of single-locus molecular monitoring when integrated with hematological assessment. Key limitations include that dPCR or NGS-SNP analysis was not performed during the case monitoring period, preventing direct quantification of low-level chimerism below STR resolution. The molecular result is therefore reported as consistent with full donor-derived hematopoiesis rather than as a quantitatively certified exclusion. A further limitation is that the PowerPlex^®^ 16 System’s biostatistical validity rests primarily on North American and Eurasian allele-frequency reference databases. Equivalent reference data for Saudi Arabian and broader Middle Eastern populations are lacking, so absolute profile identity between closely related individuals from such populations cannot be excluded with full statistical certainty, underscoring the need for population-specific reference datasets in transplant centres serving consanguineous communities.

Three clinically relevant observations emerge from this case. First, transplant laboratories may benefit from routine verification of donor–recipient STR informativity before transplantation, as fewer than two informative autosomal loci may be insufficient for reliable STR-based monitoring alone. Second, consideration may be given to implementing a predefined escalation pathway (incorporating expanded STR panels, indel or SNP assays, dPCR, and/or NGS-based chimerism analysis) before transplantation, with technology selection guided by local availability and clinical risk. Third, chimerism reports issued under impaired informativity should be interpreted cautiously and in conjunction with longitudinal hematological recovery and clinical assessment. Adoption of such practices may improve analytical robustness and patient safety, particularly in regions where consanguinity is prevalent.

## Data Availability

The datasets presented in this study can be found in online repositories. The names of the repository/repositories and accession number(s) can be found in the article/Supplementary material.
