# Supplementary material for: Case Report: Similar STR profiles in non-twin siblings complicating chimerism analysis after allogeneic hematopoietic stem cell transplantation
Source: Front Med (Lausanne). 2026 Jun 16;13:1863294. doi: 10.3389/fmed.2026.1863294 (PMC13314465; doi:10.3389/fmed.2026.1863294)
Supplement: Supplementary file 1 [file Data_Sheet_1.pdf]

Table. Clinical timeline of the episode of care organized as a milestone table (CARE guidelines).

Time Point	Category	Clinical Events, Interventions, and Findings	Key Laboratory / Molecular Results
**Initial Presentation (Age 3 years)**	**Presentation and Diagnosis**	**Presentation:**<br>• **Chief complaint:** Thrombocytopenia, easy bruising, and gingival bleeding.<br>• **Neonatal history:** Low birth weight, poor feeding.<br>• **Physical examination:** Failure to thrive, short thumbs, café-au-lait macules, soft systolic murmur.<br>• **Imaging:** Ectopic solitary left kidney with preserved function (geometric mean GFR 63 mL/min).<br>**Diagnosis:**<br>• **Bone marrow biopsy:** Hypocellularity without excess blasts; reduced megakaryocytes.<br>• **Flow cytometry & cytogenetics:** No clonal evolution.<br>• **Chromosomal fragility test:** Positive (DEB/MMC-induced breaks).<br>• **Whole-exome sequencing:** Homozygous pathogenic splice-site variant FANCA c.2778+1G>A (intron 28).<br>• **Confirmed diagnosis:** Fanconi anemia (FANCA subtype).	**Initial lab results:**<br>**WBC** 10.2 $\times 10^9$/L<br>**ANC** 4.8 $\times 10^9$/L<br>**Hb** 11.5 g/dL<br>**PLT** 34 $\times 10^9$/L<br>**MCV** 103 fL<br>**HbF** 35.6%<br>**HbA** 63.0%<br><br>Cytogenetics: Normal No clonal evolution.<br>Chromosomal fragility: Positive<br>FANCA c.2778+1G>A (homozygous)
**~1 Month Pre-Transplant**	**Pre-Transplant Evaluation**	• **Marrow failure:** Progressive cytopenias documented across multiple measurements.<br>• **Donor identification:** HLA-matched non-twin sibling donor confirmed after extended family HLA typing.<br>• **Conditioning plan:** Fludarabine-based reduced-intensity conditioning regimen with standard GvHD prophylaxis selected.	**Pre-transplantation lab results (mean):**<br>**WBC** 0.96 $\times 10^9$/L<br>**ANC** 0.22 $\times 10^9$/L<br>**Hb** 10.14 g/dL<br>**HCT** 28.95%<br>**PLT** 45.17 $\times 10^9$/L
**Pre-Transplant STR Profiling**	**Molecular Assessment**	• **STR multiplex platforms:** PowerPlex® 16 System and PowerPlex® ESI 16 Fast System (run in parallel on independently collected donor and recipient bone marrow specimens).<br>• **Informativity assessment:** 14 of 15 autosomal STR loci identical between donor and recipient. **Only informative locus:** D13S317, recipient alleles 8,11; donor alleles 11,12.<br>• **Sample identity verified:** Independent donor specimen excludes sample exchange and platform artifact.	14/15 loci: Concordant Informative locus: D13S317 only<br>**Recipient:** 8,11<br>**Donor:** 11,12
**Day 0**	**Therapeutic Intervention**	• **Procedure:** Allogeneic bone marrow transplantation from the HLA-matched non-twin sibling donor.<br>• **Conditioning regimen:** Fludarabine-based reduced-intensity conditioning.<br>• **GvHD prophylaxis:** Standard institutional protocol for pediatric Fanconi anemia.<br>• **Tolerability:** Conditioning tolerated without unexpected toxicity; no adverse events required modification of the planned regimen.	No unexpected adverse events
**Day +13**	**Neutrophil Engraftment**	• **Neutrophil engraftment achieved:** ANC ≥ 0.5 $\times 10^9$/L sustained for three consecutive days. Hematological recovery thereafter progressed without interruption.	**ANC** ≥ 0.5 $\times 10^9$/L (3 consecutive days)<br>Engraftment criterion met on schedule
**Days +18–25 (approx.)**	**Platelet Engraftment**	• **Platelet engraftment achieved:** Transfusion independence with PLT ≥ 20 $\times 10^9$/L for seven consecutive days. Sustained trilineage hematopoietic recovery established.	**PLT** ≥ 20 $\times 10^9$/L (7 consecutive days)<br>Transfusion independence achieved
**Day +352**	**Chimerism Monitoring #1**	• **Post-transplant STR analyses (both platforms):** All 15 autosomal STR loci concordant with donor genotype. **D13S317:** Recipient-specific allele 8 absent; donor alleles 11,12 present.<br>• **Interpretation:** Findings consistent with donor-derived hematopoiesis (within the analytical limitations of single-locus informativity).	15/15 loci: Donor pattern D13S317: Allele 8 absent 11,12 present<br>**Consistent with full donor chimerism**
**Day +372**	**Chimerism Monitoring #2**	• **Confirmatory post-transplant STR analysis (both platforms):** Identical findings to Day +352. **D13S317:** Donor genotype 11,12 confirmed; recipient allele 8 remains absent.<br>• **Cross-platform concordance:** Superimposable electropherogram peak heights and allelic positions on both multiplex systems.	15/15 loci: Donor pattern (confirmed) D13S317: Allele 8 absent 11,12 confirmed **Full donor pattern confirmed on both platforms**
**Day +1376 (Long-term Follow-up)**	**Outcome**	• **Hematopoiesis:** Stable trilineage hematopoiesis maintained.<br>• **Graft status:** No evidence of graft failure, relapse, or secondary clonal evolution.<br>• **Adverse events:** None attributed to the chimerism monitoring strategy.<br>• **Patient status:** Age-appropriate developmental and educational activities resumed; family reports overall satisfaction with post-transplant care and monitoring.	ANC, PLT, Hb: Within normal ranges (see Supp. Table S1)<br>**Outcome:** Durable donor-derived hematopoiesis

Abbreviations: ANC, absolute neutrophil count; GFR, glomerular filtration rate; GvHD, graft-versus-host disease; Hb, hemoglobin; HbA, adult hemoglobin; HbF, fetal hemoglobin; HCT, hematocrit; HLA, human leukocyte antigen; MCV, mean corpuscular volume; PLT, platelet count; STR, short tandem repeat; WBC, white blood cell count.

Note: Timeline constructed in accordance with CARE (CAse REport) guidelines. Day 0 = date of allogeneic bone marrow infusion. Approximate dates are used where precise post-diagnosis calendar dates are not stated. Full longitudinal hematological data are provided in Supplementary Table S1.
