# Supplementary material for: Case Report: Similar STR profiles in non-twin siblings complicating chimerism analysis after allogeneic hematopoietic stem cell transplantation
Source: Front Med (Lausanne). 2026 Jun 16;13:1863294. doi: 10.3389/fmed.2026.1863294 (PMC13314465; doi:10.3389/fmed.2026.1863294)
Supplement: Supplementary file 2 [file Table_1.docx]

**Supplementary Table S1. Early and Late Hematological Recovery following allogeneic HSCT**

| **Day post- HSCT** | **ANC (x10^9^/L)** | **PLT (x10^9^/L)** | **Hb (g/dL)** | **HCT (%)** | **RBC (x10^12^/L)** |
| --- | --- | --- | --- | --- | --- |
| 0 | 0.59 | 81 | 9.2 | 25.6 | 3.4 |
| 1 | 2.01 | 64 | 9.2 | 24.6 | 3.2 |
| 2 | 3.47 | 43 | 9 | 25.8 | 3.4 |
| 3 | 2.79 | 36 | 9.2 | 26.5 | 3.4 |
| 4 | 1.51 | 105 | 7.5 | 23.2 | 2.8 |
| 5 | 3.35 | 97 | 7.4 | 21.3 | 2.7 |
| 6 | 2.23 | 61 | 7.5 | 21.2 | 2.7 |
| 7 | 3.18 | 70 | 7.5 | 20.8 | 2.6 |
| 8 | 2.85 | 77 | 7.7 | 22.6 | 2.8 |
| 9 | 4.29 | 67 | 7.6 | 21.6 | 2.7 |
| 10 | 2.93 | 67 | 8.9 | 25.9 | 3.2 |
| 11 | 2.98 | 60 | 8.2 | 23.2 | 2.8 |
| 12 | 4.61 | 76 | 8.7 | 24 | 2.8 |
| 13 | 3.97 | 73 | 8.8 | 25 | 2.8 |
| 14 | 2.78 | 67 | 8.9 | 25.9 | 2.9 |
| 15 | 2.74 | 66 | 9.1 | 26.1 | 2.8 |
| 16 | 2.35 | 64 | 9.4 | 27.1 | 2.9 |
| 17 | 3.64 | 73 | 10.4 | 30.5 | 3.2 |
| 18 | 4.5 | 73 | 10.7 | 31 | 3.3 |
| 25 | 5.38 | 144 | 13 | 39.2 | 3.7 |
| 88 | 5.83 | 312 | 14.9 | 45.8 | 4.9 |
| 89 | 7.89 | 257 | 14.4 | 44.3 | 4.7 |
| 101 | 7.36 | 351 | 15.4 | 46.2 | 5.2 |
| 114 | 5.42 | 367 | 13.9 | 43.5 | 4.7 |
| 116 | 3.61 | 311 | 13.1 | 40.6 | 4.4 |
| 120 | 4.22 | 317 | 13.7 | 43.4 | 4.7 |
| 121 | 4.42 | 295 | 13.8 | 43.1 | 4.7 |
| 122 | 4.51 | 283 | 13.1 | 40.3 | 4.4 |
| 123 | 4.15 | 281 | 13.1 | 39.6 | 4.4 |
| 124 | 6.03 | 303 | 14.4 | 44.2 | 4.8 |
| 125 | 4.75 | 282 | 12.8 | 37.9 | 4.3 |
| 126 | 4.41 | 293 | 12.3 | 38.9 | 4.2 |
| 127 | 4.31 | 284 | 12.4 | 39.3 | 4.3 |
| 128 | 4.07 | 309 | 13.3 | 41.1 | 4.5 |
| 129 | 5.17 | 330 | 13.5 | 41.6 | 4.6 |
| 130 | 4.5 | 416 | 12.6 | 38.6 | 4.4 |
| 131 | 6.02 | 361 | 12.8 | 37.5 | 4.2 |
| 134 | 6.8 | 351 | 12.3 | 38.3 | 4.1 |
| 137 | 4.04 | 397 | 13.7 | 41.6 | 4.6 |
| 144 | 3.94 | 398 | 14.1 | 42.8 | 4.6 |
| 146 | 2.39 | 349 | 12.4 | 36 | 4.1 |
| 147 | 1.64 | 363 | 11.5 | 35.4 | 4 |
| 148 | 1.02 | 402 | 12.3 | 38.7 | 4.3 |
| 149 | 0.97 | 454 | 12.6 | 39.2 | 4.4 |
| 150 | 0.57 | 429 | 12.4 | 37.1 | 4.1 |
| 151 | 0.73 | 459 | 13.3 | 39.4 | 4.4 |
| 159 | 3.73 | 377 | 14.2 | 36.9 | 4.9 |
| 162 | 6.19 | 309 | 13.1 | 38.9 | 4.3 |
| 169 | 4.73 | 320 | 14 | 43 | 4.8 |
| 176 | 5.96 | 328 | 13.7 | 43.1 | 4.6 |
| 183 | 7.48 | 363 | 14.3 | 43 | 4.8 |
| 190 | 5.06 | 365 | 13.8 | 41.7 | 4.7 |
| 197 | 10.6 | 354 | 13.6 | 40.7 | 4.7 |
| 211 | 5.47 | 327 | 14.3 | 43.6 | 5.1 |
| 233 | 3.82 | 285 | 14 | 41.9 | 4.9 |
| 234 | 2.93 | 274 | 14.2 | 44 | 4.8 |
| 248 | 7.01 | 302 | 13.8 | 42.4 | 4.9 |
| 281 | 4.5 | 364 | 13.7 | 42.7 | 4.8 |
| 298 | 2.71 | 357 | 14.3 | 43 | 5 |
| 315 | 20.5 | 273 | 14.7 | 45.2 | 5.2 |
| 333 | 3.22 | 326 | 14 | 42.7 | 4.8 |
| 361 | 5.85 | 499 | 14.8 | 45.5 | 5.3 |
| 404 | 3.26 | 299 | 13.9 | 42.3 | 5 |
| 452 | 3.74 | 274 | 14.3 | 41.9 | 4.8 |
| 459 | 7.2 | 225 | 15.5 | 47.1 | 5.5 |
| 521 | 4.29 | 278 | 15.2 | 45 | 5.4 |
| 522 | 4.55 | 326 | 15 | 44.6 | 5.3 |
| 529 | 3.92 | 324 | 14.6 | 44.8 | 5.3 |
| 550 | 4.39 | 334 | 14.7 | 44.4 | 5.1 |
| 634 | 3.85 | 279 | 14.6 | 43.7 | 5.4 |
| 698 | 4.21 | 290 | 13.8 | 41.6 | 4.9 |
| 1096 | 5.09 | 349 | 13.9 | 40.7 | 5 |
| 1149 | 4.83 | 346 | 14.5 | 43.6 | 5.5 |
| 1120 | 5.43 | 466 | 13.6 | 41.3 | 5.4 |
| 1376 | 5.58 | 417 | 14.6 | 42.3 | 5.6 |

Early and late hematological recovery following allogeneic HSCT (daily and longitudinal ANC, platelet count, hemoglobin, hematocrit, and red blood cell count from day 0 through day +1376).
